# Research on the Influence Path of Online Consumers’ Purchase Decision Based on Commitment and Trust Theory

**DOI:** 10.3389/fpsyg.2022.916465

**Published:** 2022-08-23

**Authors:** Yan You, Yanchuan Hu, Weining Yang, Shuai Cao

**Affiliations:** ^1^Department of Business English, School of Foreign Languages, Hainan Normal University, Haikou, China; ^2^Department of Information Technology, Beijing Municipal Road and Bridge Co., Ltd., Beijing, China; ^3^Department of Hotel Management, School of Economics and Management, Hefei Normal University, Hefei, China; ^4^Department of Management, Zhijiang College, Zhejiang University of Technology, Hangzhou, China

**Keywords:** psychology, common values, online trust, promise purchase decisions, commitment and trust theory

## Abstract

Based on the theory of commitment and trust, this paper constructs an online consumer purchase decision model, empirically studies the common values, online trust, commitment and purchase decision, and explores the influence mechanism and path of online consumer purchase decision. The results show that common values of shopping platforms and online consumers have a significant positive impact on trust and commitment, among which the common value orientation of mutual trust and commitment compliance has the most significant impact, which reflects the core content of common values. Secondly, the confidentiality of buyer information, common value of ethics, accurate disclosure of information, and finally the attitude to solve problem;The establishment of online trust has a significant positive impact on commitment, and both online trust and commitment are positively related to purchase decision. In terms of research methods, the theory of commitment and trust is introduced into the study of online consumption on Chinese shopping platforms, which expands the scope of application of the theory of commitment and trust. The trust of online consumers is generated by their own shopping experience. The conclusion of this study provides a decision-making basis for the innovation of marketing model and has theoretical reference value.

## Introduction

With the promotion of Internet and smart devices, the proportion of online consumption mode in daily commodity transactions is increasing, and the scale of online consumers is also growing rapidly year by year. In recent years, scholars and e-commerce enterprises have paid extensive attention to the trust problem and the influencing factors of purchase intention promotion in online consumption. However, it is a pity that on the one hand, the quality of websites, network security and other factors have been paid close attention to, and theoretical research results are constantly emerging, on the other hand, the general lack of trust in daily transactions and the rising transaction costs year by year have become the main obstacles to the development of e-commerce.

At present, domestic scholars have little research on online consumer purchase decision, and have not yet formed a mature theoretical system, and the impact of common values on online trust and purchase decision is also vague. Shared values are the primary factor in the highest level of trust, unconditional trust (Jones and George, 1998). Without common values, it is difficult to build trust. The problem of maintaining commitment and maintaining long-term purchasing decisions is becoming increasingly prominent. Therefore, based on the theory of commitment and trust, this paper collects 551 valid survey samples, verifies the significant positive impact of shared values through structural equation model, and proposes that the establishment of shared values should be the primary development task of online shopping platforms. And then establish online trust, and ultimately achieve online consumer purchase decisions. At the same time, in terms of research methods, the theory of commitment and trust is introduced into the study of online consumption on Chinese shopping platforms, which expands the scope of application of the theory of commitment and trust.

## Literature Review and Research Hypothesis

Values influence business and personal behavior, making them the focus of management and marketing research (Hofstede, 2001). Many scholars have pointed out that shared values play an important role in building high-level trust. Among them, studies have pointed out that the decisive factor of trust is whether the two sides agree with each other’s values (Lewicki and Bunker, 1996). Cheung and Thadani described eWom as social communication content (stimuli), a new form of social communication involving both customers seeking information and customers sharing information and their response (Cheung and Thadani, 2012). This paper defines the common values as the principles followed by both sides, the confidentiality of consumer information, the attitude of dealing with problems, the degree of moral identity and the degree of openness of commodity information, that is, both sides follow the values of mutual trust and commitment; For the basic information of consumers, the shopping platform should be kept strictly confidential, and should not sell to the outside world or send promotional information without consent. When problems arise, the two sides should coordinate and solve them in time. Shopping platforms should take measures to prevent children from purchasing goods that are not conducive to growth, such as adult goods. Defective or substandard products sold at low prices should be clearly marked and the reasons for price reduction should be explained.

Online trust promotes cooperation, open communication, and commitment (Dirks and Ferrin, 2001). Trustworthiness has a significant impact on consumer behavior in online environments (Abd-Elaziz et al., 2015). In an exchange relationship, an important consideration is who is willing to exchange, and trust plays a decisive role in deciding who to exchange with (McKnight and Chervany, 2001–2002). Many studies have reported that trustworthiness has a vital impact on the exchange and exchange of information (Tseng and Hsu, 2010; Tien et al., 2019). Commitment and trust theory (Morgan and Hunt, 1994) proposes that the buyer’s trust and commitment to the seller determine the marketing effect. Trust is determined by shared values, communication behavior and opportunism. In addition, it verifies the significant relationship between commitment and trust, and concludes that trust is the key variable in the formation of commitment. The study points out that trust and commitment are the key to enterprise marketing, because trust and commitment will effectively promote both sides of the transaction to maintain long-term cooperative relationship and maintain partnership for long-term benefits. And believe that that other party will not engage in opportunistic behavior.

Based on the theory of commitment and trust, common values determine trust, and trust has a positive impact on commitment. Commitment is to maintain a long-term relationship. In the business environment, both sides of the transaction attach importance to trust and make commitments in order to obtain long-term cooperative purchase decisions. In this paper, online trust is defined as trust tendency, trust conventionality and trust technical guarantee. Commitment is defined as sense of belonging, sense of connection and transaction basis, and purchase decision is defined as purchase intention, word-of-mouth promotion intention and continuous interaction intention. And put forward the following assumptions:

H1: Shared values have a significant positive effect on online trust.
H2: Shared values have a significant positive effect on commitment.
H3: Online trust has a significant positive effect on commitment.
H4: Online trust has a significant positive effect on purchase decision.
H5: Commitment has a significant positive effect on purchase decision.

**FIGURE 1:**
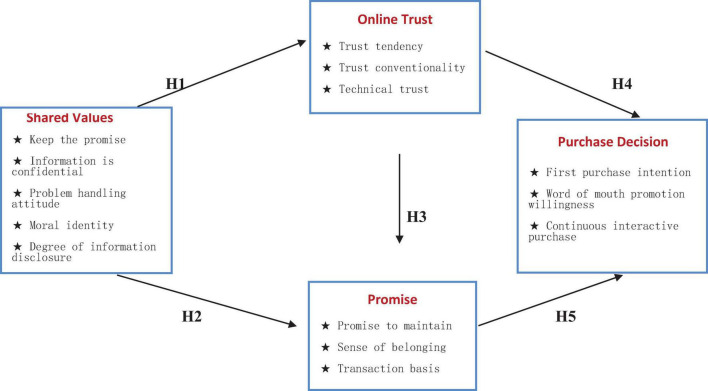
Path model of online consumer purchase decision.

To sum up, the basic structure and assumptions of the research model in this paper are shown in Figure 1.

## Model Variables and Data Collection

### Model Variables

This study uses the method of questionnaire, including two parts: the statistics of respondents’ characteristics and the dimensions of online trust model. Likert seven-point scale was used in the questionnaire. Firstly, the key variables were extracted by theoretical analysis. Seven professors who are specialized in E-commerce from the same college of Economics and management were interviewed about the theory and scale. According to their feedback, the scale was further modified and improved. In the pre-survey, Exploratory factor analysis was carried out on the collected data using SPSS23 software, and the final measurement scale was formed by deleting some items.

### Data Collection

In the pre-survey, 142 valid questionnaires were collected. In the formal survey, 551 valid questionnaires were collected, and the effective recovery rate was 73%. The respondents are mainly consumers aged 18–49 with experience in online shopping platforms, 61.4% of whom have bachelor’s degree or above, 84.6% of whom have been shopping for 2-8 years, and 60% of whom are in North China and Southwest China.

SPSS23 software was used for descriptive statistical analysis of the sample, including maximum, mean, standard deviation, skewness and kurtosis, in which the absolute value of skewness was less than 3, the absolute value of kurtosis was less than 5, and the data were normally distributed.

## Data Analysis and Hypothesis Testing

### Reliability Analysis

In order to ensure the reliability and stability of the questionnaire, SPSS was used to analyze the reliability of the questionnaire. Reliability refers to the reliability of each index and the consistency of the results obtained when the same method is used to repeatedly measure the same object. The reliability of the questionnaire is usually measured by the Alpha coefficient (Clonbach Alpha coefficient). The bigger the α coefficient is, the higher the reliability of the questionnaire is. That is, the higher the credibility and stability of the questionnaire. From Table 1, we can see that the reliability of shared values is 0.858, the reliability of online trust is 0.857, the reliability of commitment is 0.802, and the confidence of purchase decision is 0.787. Scholars (DeVellis, 1991) believe that reliability is very good at 0.80–0.90 and quite good at 0.70–0.80. Accordingly, the data collected in this questionnaire have high credibility.

**TABLE 1:** Reliability statistics and item total statistics.

Dimension	Factor	Corrected item and total correlation	Clonbach Alpha after deletion	Clone Bach Alpha
Shared values	SV1	0.742	0.813	0.858
	SV2	0.701	0.822	
	SV3	0.615	0.844	
	SV4	0.672	0.832	
	SV5	0.657	0.833	
Online trust	OT1	0.736	0.795	0.857
	OT2	0.754	0.776	
	OT3	0.702	0.826	
Promise	CO1	0.634	0.743	0.802
	CO2	0.628	0.749	
	CO3	0.68	0.695	
Purchase decision	BI1	0.628	0.71	0.787
	BI2	0.67	0.662	
	BI3	0.586	0.753	

### Principal Component Factor Analysis

First of all, factor analysis was conducted on the samples, and the method of principal component analysis was used. The KMO value of the sample data is 0.844, and the Bartlett sphericity test *P* value is 0.000, which is lower than 0.05, which meets the two conditions of factor analysis. Factor analysis is carried out on the sample, factor rotation is carried out, and the first four principal components are extracted. The results show that four common factors are extracted from the scale. The cumulative explanation of the four common factors to the total variance is 70.19%, which is more than 50%, and the factor load of each item in the corresponding latitude is more than 0.5, indicating that each item can explain the latitude well. In addition, there is no Cross. Loading phenomenon, indicating that there is a certain degree of differential validity between the dimensions. To sum up, the validity of the above scale is good. Table 2 shows the factor loading matrix after varimax rotation.

**TABLE 2:** Factor load matrix.

Dimension	Factor	Ingredients			
		1	2	3	4
Shared values	SV1	0.828	0.114	0.126	0.070
	SV2	0.804	0.091	0.101	0.055
	SV3	0.742	0.113	0.048	0.082
	SV4	0.785	0.087	0.123	−0.002
	SV5	0.765	0.095	0.127	0.082
Online trust	OT1	0.065	0.869	0.064	0.187
	OT2	0.149	0.858	0.152	0.147
	OT3	0.217	0.821	0.088	0.144
Promise	CO1	0.127	0.065	0.817	0.144
	CO2	0.120	0.091	0.797	0.187
	CO3	0.171	0.132	0.835	0.096
Purchase decision	BI1	0.078	0.127	0.123	0.819
	BI2	0.084	0.139	0.144	0.840
	BI3	0.052	0.183	0.152	0.765
Initial eigenvalue		4.737	2.231	1.616	1.243
The sum of the squares of the rotating loads	Totally	3.234	2.315	2.153	2.125
	% of variance	23.102	16.533	15.381	15.177
	Cumulative%	23.102	39.635	55.016	70.193
KMO		0.844			
Bartlett’s test for sphericity	Approximate chi-square	3,243.661			
	df	91			
	Sig.	0.000			

### Confirmatory Factor Analysis

In this study, confirmatory factor analysis was used to test the degree of fit between the actual collection data of the questionnaire scale and the factor structure model, and whether the index variables can be effectively used as latent variables. The data in Table 3 show that the average extraction variance AVE of common values, online trust, commitment and purchase decision is higher than 0.55. The results showed that the scale had good convergent validity.

**TABLE 3:** Confirmatory factor analysis.

Dimension	Factor	Standardized estimate	Composite reliability	AVE
Shared values	SV1	0.817	0.86	0.55
	SV2	0.759		
	SV3	0.675		
	SV4	0.741		
	SV5	0.725		
Online trust	OT1	0.816	0.86	0.67
	OT2	0.856		
	OT3	0.78		
Promise	CO1	0.735	0.8	0.58
	CO2	0.734		
	CO3	0.806		
Purchase decision	BI1	0.741	0.79	0.58
	BI2	0.806		
	BI3	0.688		

After verifying the reliability and validity of each measurement scale, the paper uses AMOS22 structural equation software to test the path hypothesis in the model. Where (χ^2^/DF = 1.453, RMR = 0.052, RMSR = 0.028, GFI = 0.974, AGFI = 0.962, NFI = 0.968, IFI = 0.99, CFI = 0.99, and RMSEA = 0.029. The (χ^2^/DF, RMSR, and RMSEA of the evaluation model are close to the ideal value, and other fitting indicators fit well, so the established model is acceptable. The fitting indexes of the factor analysis model are shown in Table 4.

**TABLE 4:** Fit indicators of confirmatory factor analysis model.

Statistical inspection quantity	Criteria or thresholds for adaptation	Inspection result data	Model adaptation judgment
χ^2^/df	<3.00	1.453	Yes
RMR	<0.05	0.052	Approx. 0.05
RMSR	<0.05	0.028	Yes
GFI	>0.90	0.974	Approx. 0.9
AGFI	>0.90	0.962	Approx. 0.9
NFI	>0.90	0.968	Approx. 0.9
IFI	>0.90	0.99	Approx. 0.9
CFI	>0.90	0.99	Approx. 0.9
RMSEA	<0.08	0.029	Yes

### Structural Equation Analysis

**FIGURE 2:**
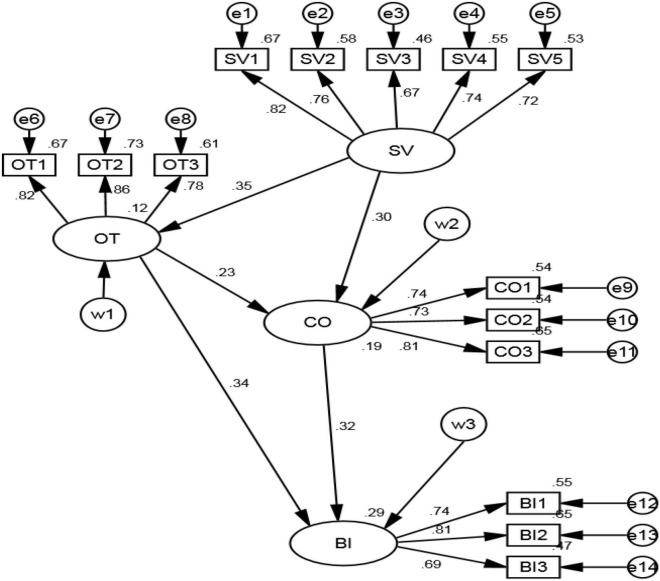
Normalized path results.

The estimation of common values, online trust, commitment and purchase decision model is mainly completed by path analysis, which mainly examines the strength and reliability of causal relationship. Import the collected data into the model and analyze it to get Figure 2.

It can be seen from Table 5 that the *P* values between the hypotheses are all less than 0.001, indicating that there is a significant correlation between them, and the standardized coefficients are all positive, which passes the hypothesis test. Shared values have a significant positive impact on online trust; Both shared values and online trust have a positive effect on commitment, and the effect of shared values is more significant. Both online trust and commitment have a positive impact on purchase decision, and commitment has a more significant impact on purchase decision. To sum up, hypotheses H1, H2, H3, H4, and H5 are verified.

**TABLE 5:** Analysis of normalized path coefficient.

Assumptions		Estimate	Standardized estimate	SE	CR	*P*	Hypothesis test results
H1	OT←SV	0.419	0.35	0.059	7.156	***	Support
H2	CO←OT	0.198	0.235	0.045	4.421	***	Support
H3	CO←SV	0.302	0.299	0.054	5.582	***	Support
H4	BI←OT	0.248	0.341	0.038	6.458	***	Support
H5	BI←CO	0.274	0.318	0.047	5.793	***	Support

***P < 0.001

## Conclusion and Prospect

Based on the theory of commitment and trust, this paper takes the shopping platform Taobao as the survey subject and the domestic online consumers as the survey object, and constructs a research model of online consumer purchase decision.

First, the theory of commitment and trust is also applicable in the context of Chinese shopping platforms. By studying the factors of common values and their positive effects on online trust, commitment and purchase decision, this paper further verifies the explanatory power of commitment trust theory and expands the applicable boundary of the theory.

Second, common values have a significant positive impact on online trust and commitment, and then affect the purchase decision of online consumption. Among them, both sides of the transaction should abide by the common values, that is, mutual trust and commitment, with the highest standardization coefficient, followed by the confidentiality of buyer information, common ethics and accurate information disclosure. Finally, the attitude of solving problems. Therefore, in order to improve consumer trust and expand sales, shopping platforms need to establish correct corporate values, adhere to mission identity, vision influence, situational infection, environmental edification, and build systematic and institutionalized values.

Third, both commitment and online trust have a significant positive impact on purchase decisions. Among them, the trust of online consumers generated by their own shopping experience has the most significant impact on purchasing decisions. Therefore, the shopping platform should first optimize the big data, according to the consumer’s consumption habits and needs, one-stop integration of information, to facilitate consumer decision-making; Innovating the market promotion mode, It even launched time-limited free activities to attract consumers to participate in the experience and generate trust. On this basis, we should increase consumer stickiness and expand consumer groups through the concept of sharing economy. Deeply structure products and brands, promote consumers to share comments, accumulate consumer interest data, and complete the closed-loop consumption data.

Fourth, for e-commerce practitioners, it is also very important to establish common values with consumers. Practitioners need understand the actual requirements of consumers, respect consumption habits, and reasonably recommend products and advertisement. Meanwhile, Practitioners try to avoid product quality problems, do a good job in customer after-sales service, protect consumer privacy, so as to establish mutual trust.

There are some limitations in this study. The subjects of this study are domestic online consumers. Future research can consider the factors of transnational online consumption, international values and cultural differences. In addition, as a new e-commerce model in the past decade, shopping platform has problems of product quality and service model, which are rooted in the differences in values between the two sides of the transaction. There is a dynamic process of common values changing with the development of society, which deserves further attention in the follow-up theoretical research. How to establish good common values is also a realistic problem to be solved urgently in Chinese society.

## Data Availability Statement

The raw data supporting the conclusions of this article will be made available by the authors, without undue reservation.

## Ethics Statement

Ethical review and approval was not required for the study on human participants in accordance with the local legislation and institutional requirements. Written informed consent from the patients/ participants or patients/participants legal guardian/next of kin was not required to participate in this study in accordance with the national legislation and the institutional requirements.

## Author Contributions

YH: data analyzing. WY: data collection. SC: draft. All authors contributed to the article and approved the submitted version.
